# The prevalence of pelvic organ prolapse and associated factors in Ethiopia: a systematic review and meta-analysis

**DOI:** 10.3389/fmed.2023.1193069

**Published:** 2023-07-05

**Authors:** Dagne Addisu, Maru Mekie, Yismaw Yimam Belachew, Alemu Degu, Natnael Atnafu Gebeyehu

**Affiliations:** ^1^Department of Midwifery, College of Health Sciences, Debre Tabor University, Debre Tabor, Ethiopia; ^2^Department of Obstetrics and Gynecology, College of Health Sciences, School of Medicine, Debre Tabor University, Debre Tabor, Ethiopia; ^3^School of Midwifery, College of Health Science and Medicine, Wolaita Sodo University, Sodo, Ethiopia

**Keywords:** pelvic organ prolapse, associated factors, Ethiopia, systematic review and meta-analysis, prevalence

## Abstract

**Background:**

Pelvic organ prolapse (POP) affects millions of women globally, with resource-limited countries, such as Ethiopia, carrying the highest burden. Previously, the prevalence of POP was estimated using seven studies. However, this study lacks generalization because of the limited number of studies and low geographical representation. In total, 12 additional primary studies were conducted after this review, and their reported prevalence was significantly variable across the studies. In addition, different new factors were reported in the primary studies. Therefore, this study aimed to update the pooled prevalence of POP and its associated factors in Ethiopia.

**Methods:**

This study was conducted in accordance with the Preferred Reporting Items for Systematic Reviews and Meta-analyses guidelines. Articles that were published between 2000 and 2023 were searched using the African Journal of Online, ScienceDirect, DOAJ, PubMed, and Google Scholar. The quality of the studies was evaluated using the modified Newcastle-Ottawa quality assessment tool. The data were extracted using Microsoft Excel and analyzed by Stata version 11. A random effect model was used to investigate the pooled prevalence of POP and its associated factors. The I^2^ test and Egger's regression test were used to detect the presence of heterogeneity and publication bias across studies, respectively.

**Result:**

A total of 21 studies met the inclusion criteria and represented the data of 14,575 women. The pooled prevalence of POP was found to be 22.70%. History of home delivery (pooled odds ratio (OR) =2.93, 95% CI =1.46, 5.91), prolonged labor (OR = 4.63, 95% CI = 2.56, 8.38), history of perineal tear (OR = 4.83, 95% CI = 2.31, 10.11), instrumental delivery (OR =3.70, 95% CI =2.01, 6.81), grand multipara (OR = 5.42, 95% CI = 4.06, 7.23), family history of POP (OR = 3.30, 95% CI = 2.07, 5.25), and carrying heavy objects (OR = 3.23, 95% CI = 2.22, 4.70) were significantly associated with POP.

**Conclusion:**

The pooled prevalence of POP was high in Ethiopia. The Ministry of Health and clinicians should emphasize counseling on modifiable risk factors and develop further prevention strategies.

## Introduction

Pelvic organ prolapse (POP) is a gynecological disorder that affects millions of women globally (1). It is an abnormal descent of the pelvic organs mainly the bladder, uterus, rectum, and small intestine from their natural position into or outside the vagina due to a defect in the pelvic supporting structures (2–4).

Determining the actual prevalence of POP is challenging because of the varying definitions and diagnosis methods in the literature as well as in clinical practice (5). Globally, the magnitude reaches up to 15% with symptoms-based diagnosis and 64.6% with clinically based diagnosis (5, 6). Evidence revealed that the prevalence of POP was 27.5% in Uganda (7), 8% in Nepal (8), 10.3% in Pakistan (2), 15.6% in Bangladesh (9), and 9.6% in China (10).

Various factors are responsible for the occurrence of POP. Some of these factors are older age, high parity, obesity, early age at first delivery, forceps delivery, the prolonged second stage of labor, performing heavy work, chronic constipation, and trauma to the vagina (2).

Pelvic organ prolapse frequently affects women's physical, sexual, social, and psychological functions (4, 7, 11, 12). It has a significant economic burden due to impaired daily activities and decreased productivity (10). The burden of POP is significantly higher in resource-limited countries because of the challenging socioeconomic and institutional factors (13).

In Ethiopia, the burden of POP is significantly higher due to the high fertility rate, early marriage accompanied by early childbirth that results in several vaginal deliveries during a lifetime, the higher rate of home delivery, and the heavy physical burdens of daily work (14, 15). A systematic review and meta-analysis revealed that the pooled prevalence of POP was found to be 23.52% (16).

Pelvic organ prolapse severely affects women's lives in Ethiopia due to delays or poor health-seeking behavior caused by the fear of disclosing the problem to avoid losing social value or stigma (5). Approximately 67.7% of POP patients with an advanced stage develop depression (17), and approximately 47.0% of women had sexual dysfunction in Ethiopia (18). Evidence also revealed that 10.6% of all gynecologic hospital admissions and 43.8% of all gynecologic operations were due to POP (19).

Previously, a meta-analysis was done to assess the pooled prevalence of POP in Ethiopia using seven studies in 2020 (16). However, this study lacks generalization because of the limited number of studies and limited geographical representation. In total, 12 primary studies were conducted after this review in different areas of Ethiopia, and their reported prevalence was significantly variable across studies with a range of 8.9% to 66.9% (20, 21). Furthermore, additional factors, such as a family history of POP, carrying heavy objects, history of pronged labor, instrumental delivery, and perineal tear on the last childbirth, were reported in recent studies. Thus, updated and comprehensive evidence is important to identify high-risk patients and develop prevention strategies and management modalities. Therefore, this systematic review and meta-analysis study aimed to update the pooled prevalence and associated factors of POP in Ethiopia. The result of this study may improve clinicians' and patients' understanding of the individual risk factors for POP.

## Methods

### Data source

This systematic review and meta-analysis were carried out in accordance with the Preferred Reporting Items for Systematic Reviews and Meta-analyses (PRISMA) guideline (22) (Supplementary Table S1). The protocol for this systematic review was not registered in PROSPERO. All relevant studies related to POP in Ethiopia were searched in the following databases: African Journals Online (AJOL), ScienceDirect, Direct Open Access Journal (DOAJ), PubMed, and Google Scholar. We also examined each study's reference list to identify missed studies.

### Literature search strategies

The following keywords were used to establish searching strategies: prevalence, burden, proportion, POP, uterovaginal prolapse, pelvic organ dysfunction, associated factors, determinants, predictors, and Ethiopia. To create search strategies, keywords were connected by using Boolean operators (“OR” or “AND”). We applied the following search strategy for the PubMed database: (“epidemiology”[Subheading] OR “epidemiology”[All Fields] OR “magnitude”[All Fields] OR “Proportion”[All Fields] OR “prevalence”[All Fields] OR “prevalence”[MeSH Terms]) AND (“pelvic organ prolapse”[MeSH Terms] OR (“pelvic”[All Fields] AND “organ”[All Fields] AND “prolapse”[All Fields]) OR “pelvic organ prolapse”[All Fields]) AND (“associated” [All Fields] AND “factors” [All Fields] OR “determinants”[All Fields] OR “predictors” [All Fields]) AND (“Ethiopia”[MeSH Terms] OR “Ethiopia”[All Fields]). Regarding google scholar search, we used the following search strategy: (“prevalence” or “burden” or “proportion”) and “pelvic organ prolapse” and (“associated factors” or “determinants” or “predictors”) and “Ethiopia”. For African Journals Online, the search strategy was as follows: (prevalence or burden or proportion) and pelvic organ prolapse and (associated factors or determinants) and Ethiopia. Regarding ScienceDirect, the search strategy was “Pelvic organ prolapse and associated factors and Ethiopia”, whereas the search strategy for DOAJ was “Pelvic organ prolapse and associated factors and Ethiopia” (Supplementary Table S2).

### Selection and exclusion criteria

To determine searching strategies and eligibility criteria, we followed the condition, context, and population (CoCoPop) approach. This study included articles that fulfilled the following criteria: (1) studies conducted in Ethiopia; (2) studies that were published between 2000 and 2023; (3) all observational studies (cross-sectional, case–control, and cohort studies); and primary studies that investigate either the prevalence or associated factors of POP or both. Regarding exclusion criteria, we excluded studies with different outcomes of interest and qualitative research from this study.

### Measurement of outcome

This meta-analysis study has two outcomes, namely, the prevalence of POP and its associated factors. Any prolapse reported by primary studies, such as uterine prolapse, vaginal prolapse, enterocele, cystocele, or rectocele, was considered a POP and included in this meta-analysis. In the included primary studies, POP was diagnosed either subjectively by self-reported symptomatic prolapses or objectively measured prolapses by qualified specialists.

### Study selection and quality assessment

All retrieved studies were exported to the Endnote reference manager program, version 7, and assessed by using the criteria such as title, abstract, full-text availability, duplication, and study quality. The modified Newcastle-Ottawa quality evaluation tool was used to check the quality of studies (23). DA and MM assessed the quality of the studies independently. Disagreements between the authors at the time of the quality evaluation were settled by a third author (YB). Finally, high-quality articles with at least 7 points out of 10 possible points for cross-sectional studies and case–control studies were included in this meta-analysis (Supplementary Table S3).

### Data extraction

Two authors (DA and AD) extract pertinent data by using a data extraction form in Microsoft Excel. This form contains the name of the first author, study setting or region, publication year, study design, sampling design, sample size, prevalence of POP, and adjusted odds ratio (AOR) with a 95% confidence interval for significant risk factors.

### Data analysis

A random effect model was used to calculate the pooled prevalence of POP and its associated factors in Ethiopia (24–26). The I-square test was used to detect the presence of heterogeneity across the studies, whereas the funnel plot and Egger's regression test were used to check the presence of small study effects or publication bias (27). To handle the source of heterogeneity, sub-group and sensitivity analyses were conducted. Finally, the Trim and fill analysis was conducted to deal with publication biases.

## Result

### Search outcomes

**Figure 1:**
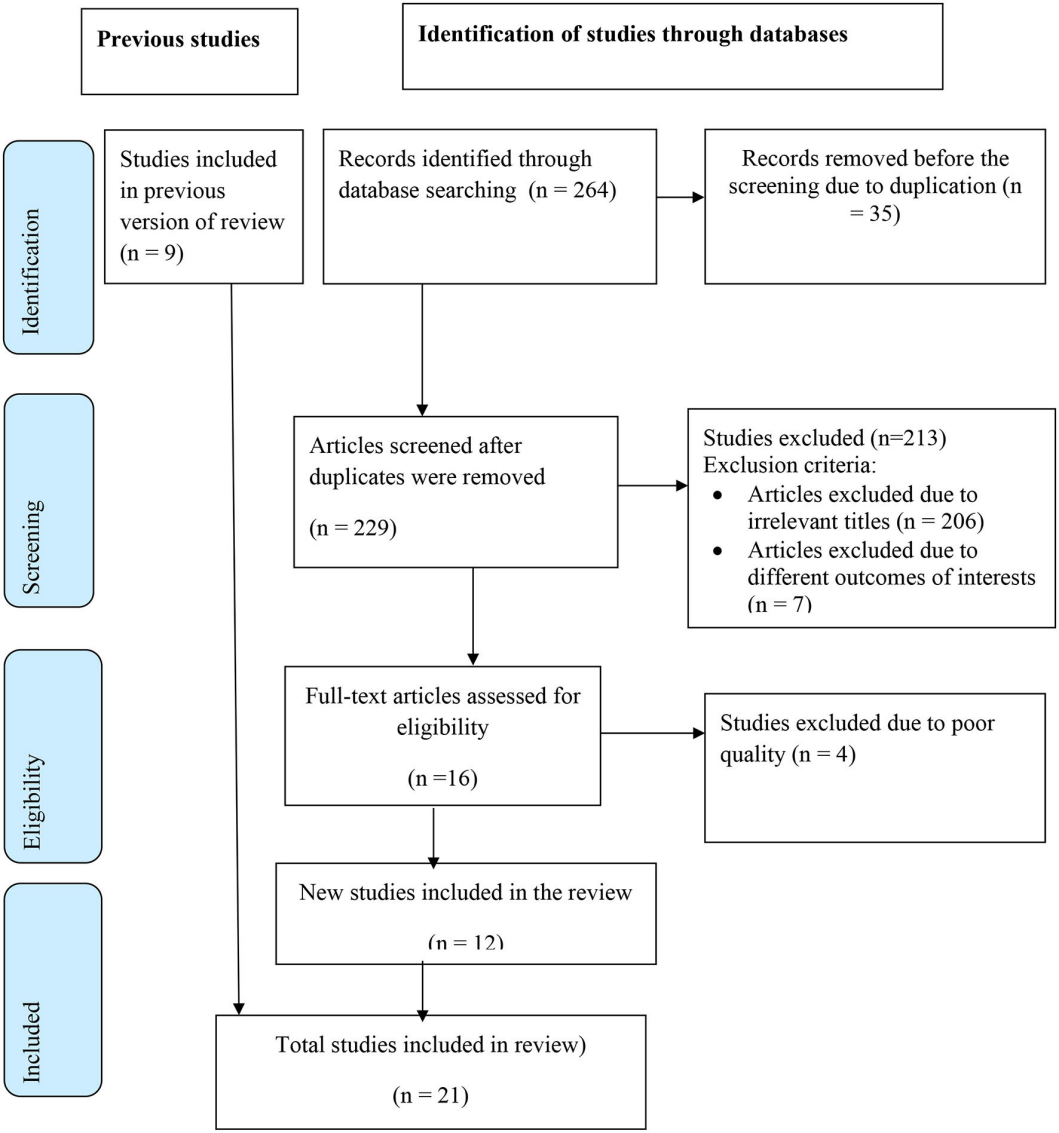
Schematic presentation of study selection to estimate the pooled prevalence of POP and its associated factors in Ethiopia.

A total of 264 articles were retrieved from different databases (PubMed = 156, Google Scholar = 22, DOAJ = 10, ScienceDirect = 69, and AJOL = 7). Out of these, 35 articles were removed from screening due to duplication. Then a total of 229 articles were screened by titles, inclusion, and exclusion criteria. In total, 206 studies were removed due to irrelevant titles; seven studies were excluded due to different outcomes of interest; and four studies were excluded due to poor quality scores based on the modified Newcastle-Ottawa quality evaluation tool. Finally, 21 articles were included to estimate the pooled prevalence of POP and its associated factors in Ethiopia (Figure 1).

### Characteristics of included studies

A total of 21 studies were included in this systematic review and meta-analysis. Out of these, eight studies were case–control studies (13, 21, 28–33) and 13 studies were cross-sectional studies (15, 20, 21, 34–43). Regarding sampling design, five studies were community-based studies and the remaining were facility-based studies. Five regions namely, South Nation, Nationalities, and Peoples' Region (SNNPR), Sidama, Amhara, Harari, and Oromia region, and one administrative city were represented in this study (Table 1).

**Table 1:** Characteristics of the included studies to estimate the pooled prevalence of POP in Ethiopia.

**Author**	**Publication year**	**Region**	**Study design**	**Sampling design**	**Sample size**	**Prevalence (%)**
Asfaw et al. (28)	2023	SNNPR	Case–control	Facility-based	369	N/A
Bezabih et al. (13)	2022	Sidama	Case–control	Facility-based	231	N/A
Zinash et al. (29)	2015	SNNPR	Case–control	Facility-based	318	N/A
Ayana et al. (20)	2022	SNNPR	CS	Facility-based	275	8.9
Nurye et al. (30)	2021	Amhara	Case–control	Facility-based	348	N/A
Ayalnesh et al. (31)	2016	Amhara	Case–control	Facility-based	370	N/A
Menur et al. (32)	2022	SNNPR	Case–control	Facility-based	348	N/A
Andualem Henok (38)	2017	SNNPR	CS	Community-based	422	13.3
Dawit et al. (15)	2022	Harari	CS	Facility-based	458	10.58
Abdek et al. (39)	2022	Harari	CS	Facility-based	387	10.1
Merega et al. (35)	2018	Oromia	CS	Community-based	3,432	9.5
Tadesse et al. (36)	2020	Amhara	CS	Community-based	880	46.7
Haymanot et al. (37)	2021	Amhara	CS	Facility-based	424	37.6
Eskedar et al. (40)	2021	SNNPR	CS	Community-based	542	25.5
Kassahun et al. (42)	2021	SNNPR	CS	Facility-based	257	10.5
Abebe et al. (41)	2022	SNNPR	CS	Community-based	422	5.9
Gamachis et al. (33)	2022	Oromia	Case–control	Facility-based	258	N/A
Zelalem (43)	2021	Addis Ababa	CS	Facility-based	3,949	12.8
Tadios et al. (44)	2021	SNNPR	Case–control	Facility-based	401	N/A
Menur et al. (34)	2012	Oromia	CS	Facility-based	143	40.7
Dabash Gezu (21)	2015	SNNPR	CS	Facility-based	341	66.9

CS, cross-sectional; N/A, not applicable; SNNPR, South Nation, Nationalities, and Peoples region.

### The pooled prevalence of POP in ethiopia

**Figure 2:**
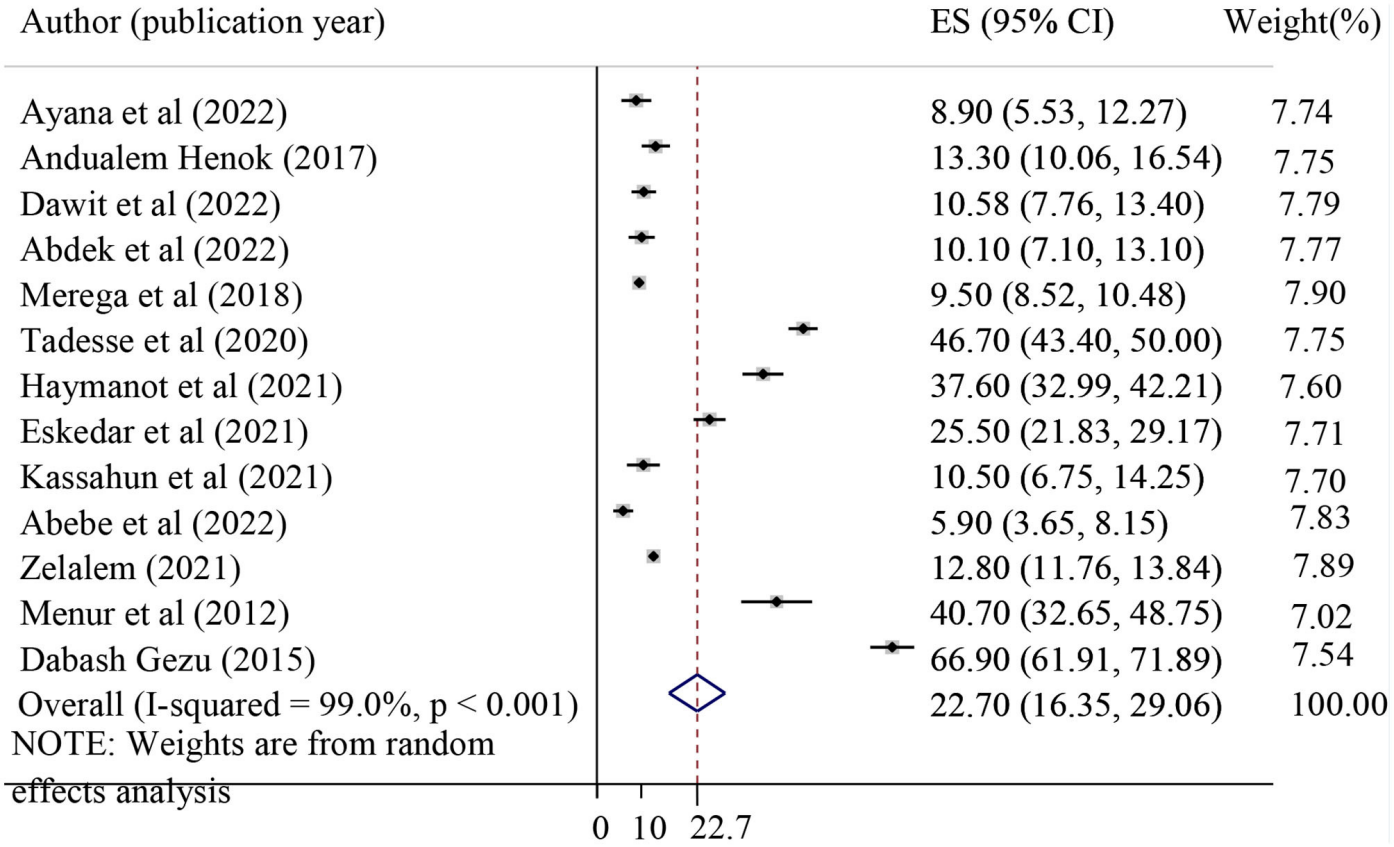
Forest plot showing the pooled prevalence of pelvic organ prolapse in Ethiopia. ES, effect size (equivalent to prevalence).

A total of 13 studies were included to determine the pooled prevalence of POP. The pooled prevalence of POP in Ethiopia was found to be 22.70% with 95%CI (16.35, 29.06). A random effect model was applied to determine the pooled prevalence of POP (Figure 2).

### Sub-group analysis

Heterogeneity was detected across the studies (I-squared = 99.0%, *p* ≤ 0.001). Therefore, sub-group analysis was performed to identify the source of heterogeneity by using different characteristics such as study region, sampling design, and sample size. In the sub-group analysis, the pooled prevalence of POP was the highest in the Amhara region (42.30%) and the lowest in the Harari region (10.36%) (Table 2).

**Table 2:** Pooled prevalence of pelvic organ prolapse for sub-group analysis by using different characteristics.

**Variable**	**Characteristics**	**The pooled prevalence of POP (95%CI)**	***I*^2^ and *P*-value**
Sampling design	Facility-based	24.49 (14.68, 34.29)	(98.8%, *p ≤* 0.001)
	Community-based	20.12 (8.09, 32.15)	(99.2%, *p ≤* 0.001)
Sample size	>500	22.51 (12.15, 32.87)	(98.8%, *p ≤* 0.001)
	≤ 500	23.47 (13.32, 33.62)	(99.4%, *p ≤* 0.001)
Region	Amhara	42.30 (33.39, 51.21)	(89.9%, p = 0.002)
	Oromia	24.83 (5.74, 55.40)	(98.2%, *p ≤* 0.001)
	Harari	10.36 (8.30, 12.41)	(0, *p* = 0.819)
	Addis Ababa	12.80 (11.76, 13.84)	N/A
	SNNPR	70.34 (63.87, 76.81	(88.5%, *p ≤* 0.001)

N/A, not applicable; POP, pelvic organ prolapse; SNNPR, South Nation; Nationalities, and Peoples region.

### Sensitivity analysis

Sensitivity analysis was performed to check the influence of individual studies on the pooled prevalence of POP. However, there were no individual studies that influence the pooled prevalence of POP (Supplementary Table S4).

### Small study effect/publication bias

**Figure 3:**
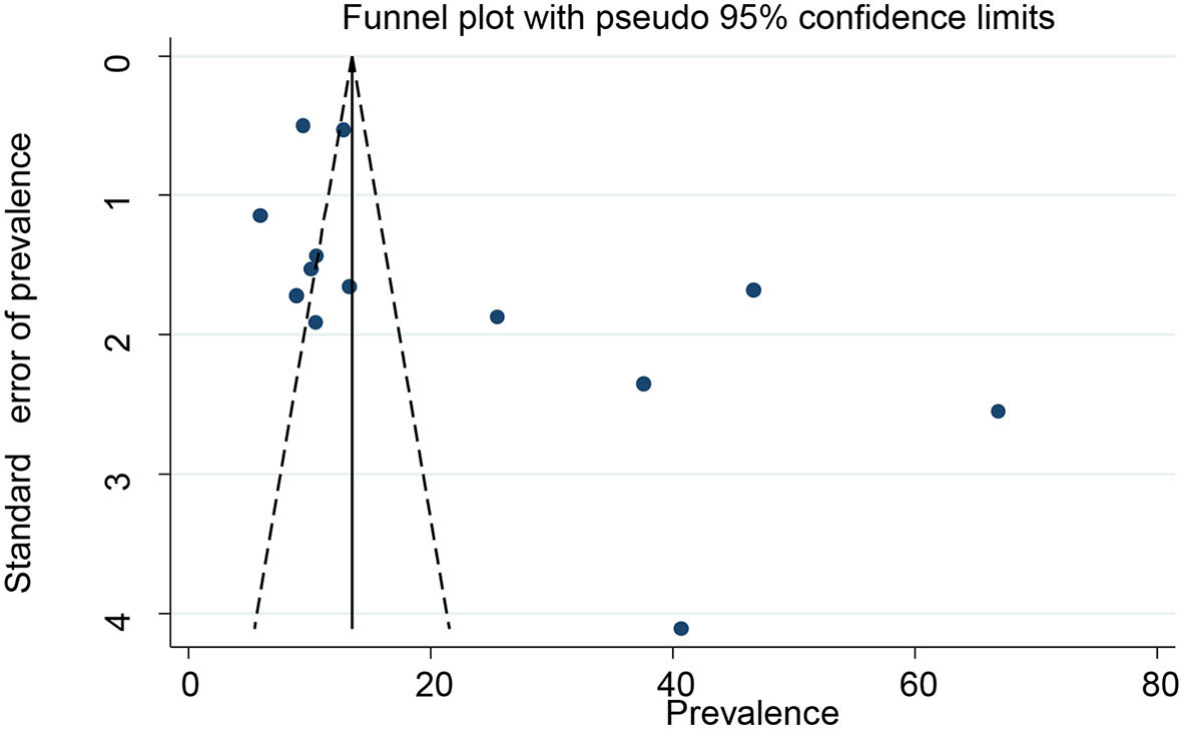
Funnel plots of primary studies for pelvic organ prolapse in Ethiopia.

**Figure 4:**
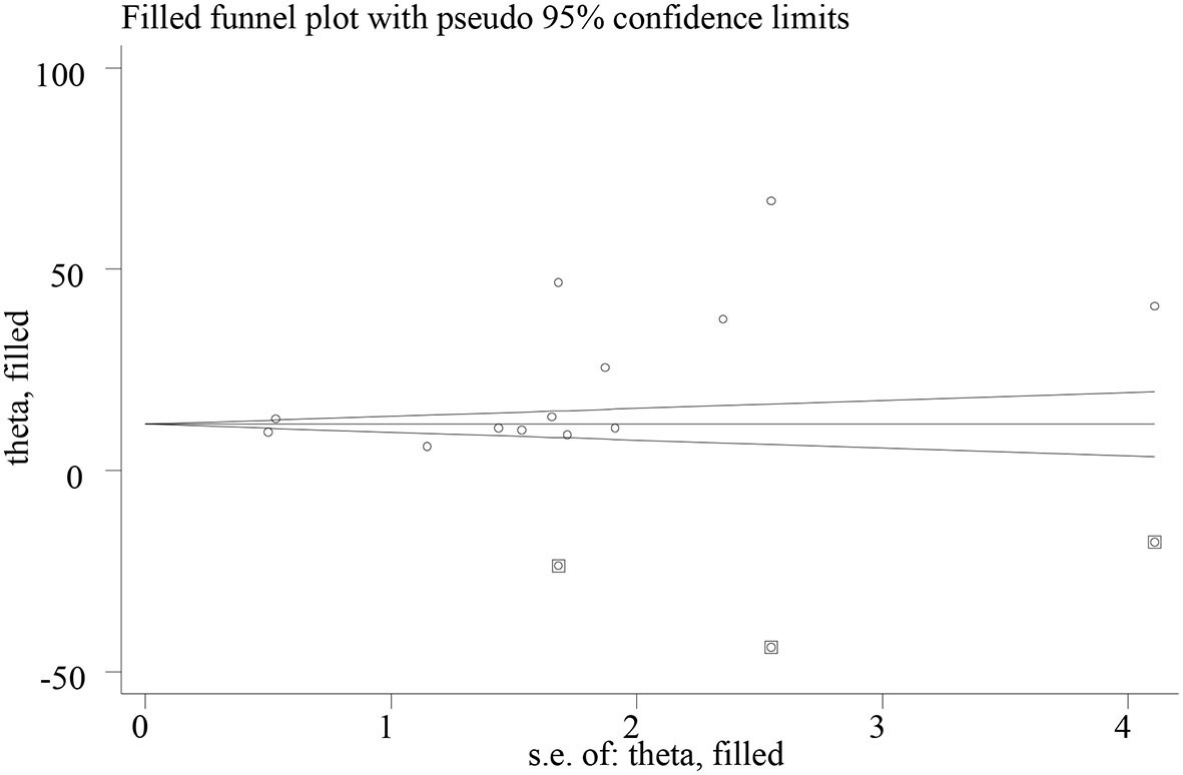
Funnel plots showing the results of trim-and-fill analysis for correcting small study effects. The rectangle dots reflect the missing studies imputed after adjustment for publication bias using the trim-and-fill approach, and the circular dots represent the identified studies included in the meta-analysis.

The presence of small study effects or publication bias across the studies was evaluated using the funnel plot and Eggers test. There was an asymmetric distribution on the funnel plot, which indicates the presence of publication bias across the studies (Figure 3). In addition, Egger's regression test found the presence of a small study effect or publication bias across the studies (*p*-value of 0.038). We performed a “trim-and-fill” analysis, which conservatively imputes three hypothetical unpublished studies to adjust for the effect of publication bias and produce a symmetrical funnel plot. The pooled prevalence of POP after incorporating the hypothetical studies was 13.33, with a 95% CI of 5.78 and 20.87 (Figure 4).

### Factors associated with POP

#### The association between the history of home delivery and POP

**Figure 5:**
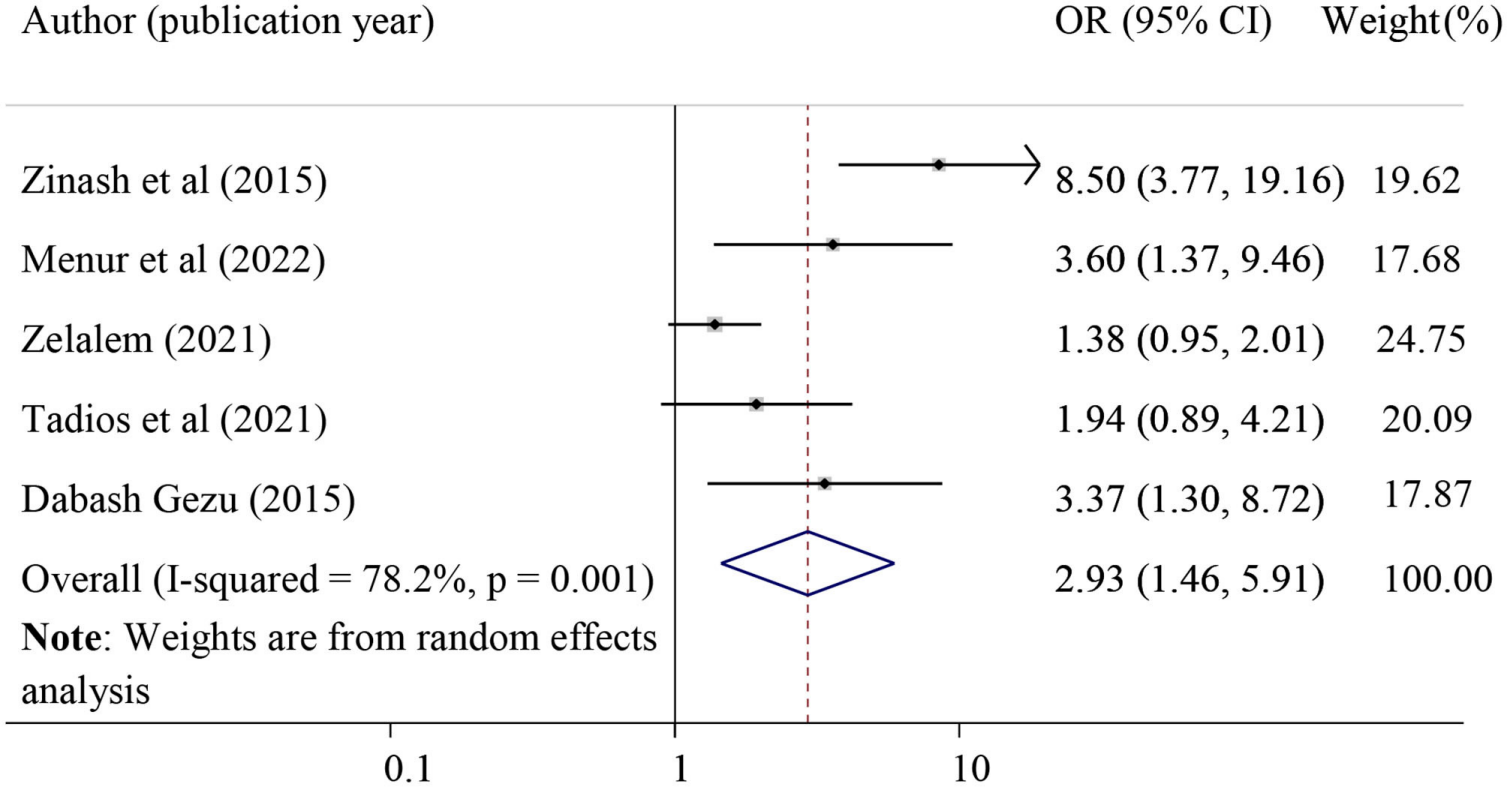
Forest plot for the association of previous history of home delivery and pelvic organ prolapse. OR, odds ratio.

The effect of home delivery on POP was examined by using five primary studies (21, 29, 32, 43, 44). The result of this study revealed that the previous history of home deliveries was significantly associated with POP. Those mothers with a previous history of home delivery were 2.93 times more likely to develop POP as compared to those mothers who had institutional delivery (pooled odds ratio = 2.93, 95% CI = 1.46, 5.91) (Figure 5).

#### The effect of the history of perineal tears on POP

**Figure 6:**
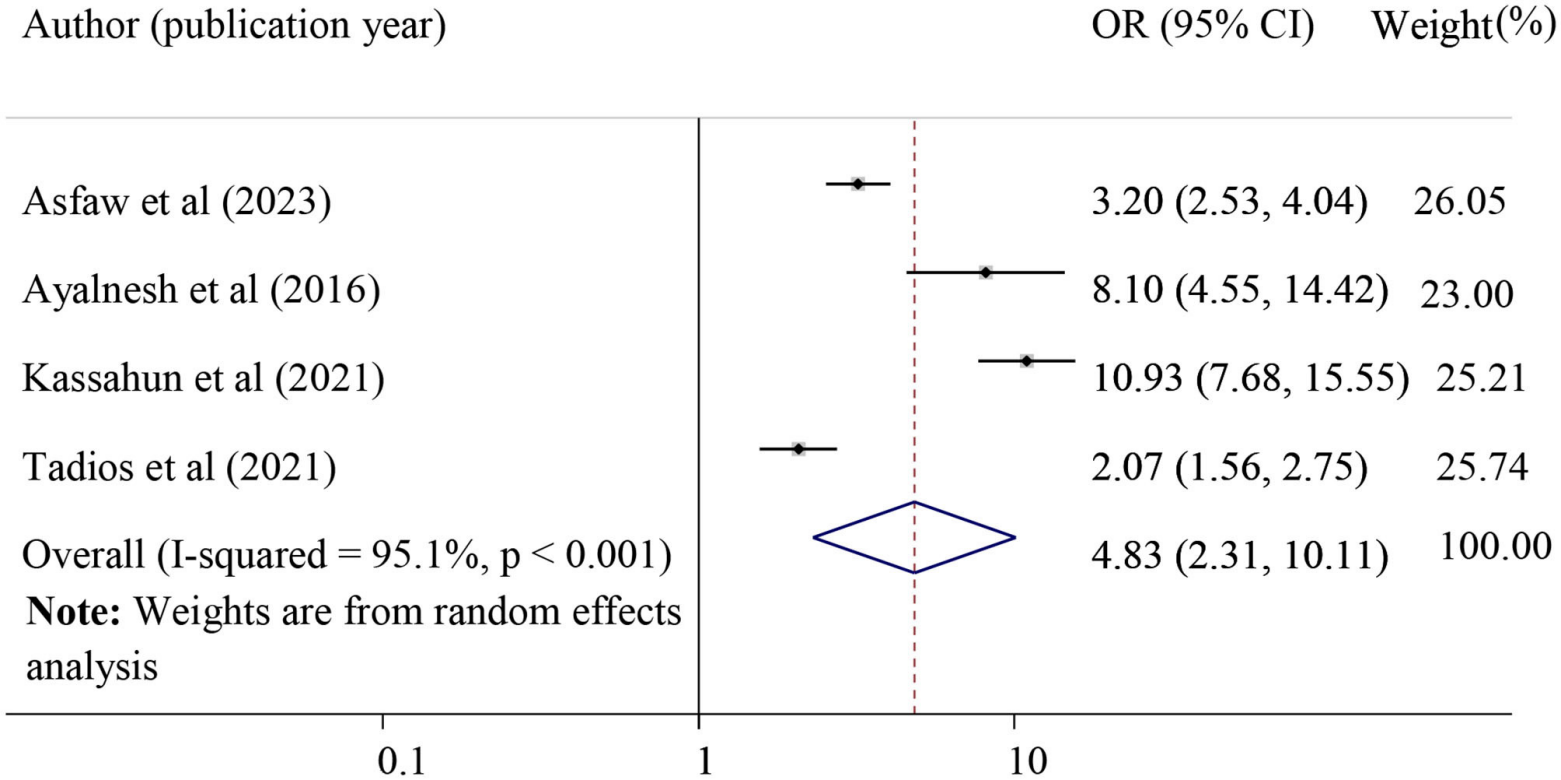
Forest plot of the association between a history of perineal tear and pelvic organ prolapse. OR, odds ratio.

The effect of the history of perineal tears on POP was evaluated by using four primary studies [28, 31, 42, and 44]. The result of this meta-analysis revealed that a history of perinatal tears was significantly associated with POP. Those mothers who had a history of perineal tears were 4.83 times more likely to develop POP as compared to their counterparts (pooled odds ratio = 4.83, 95% CI = 2.31, 10.11). Included studies defined perineal tear when there was an injury to the skin and associated soft tissue structures, primarily the superficial perineal skin, the vagina, or other areas of the vulva, such as the labia (Figure 6).

#### The association between family history and POP

**Figure 7:**
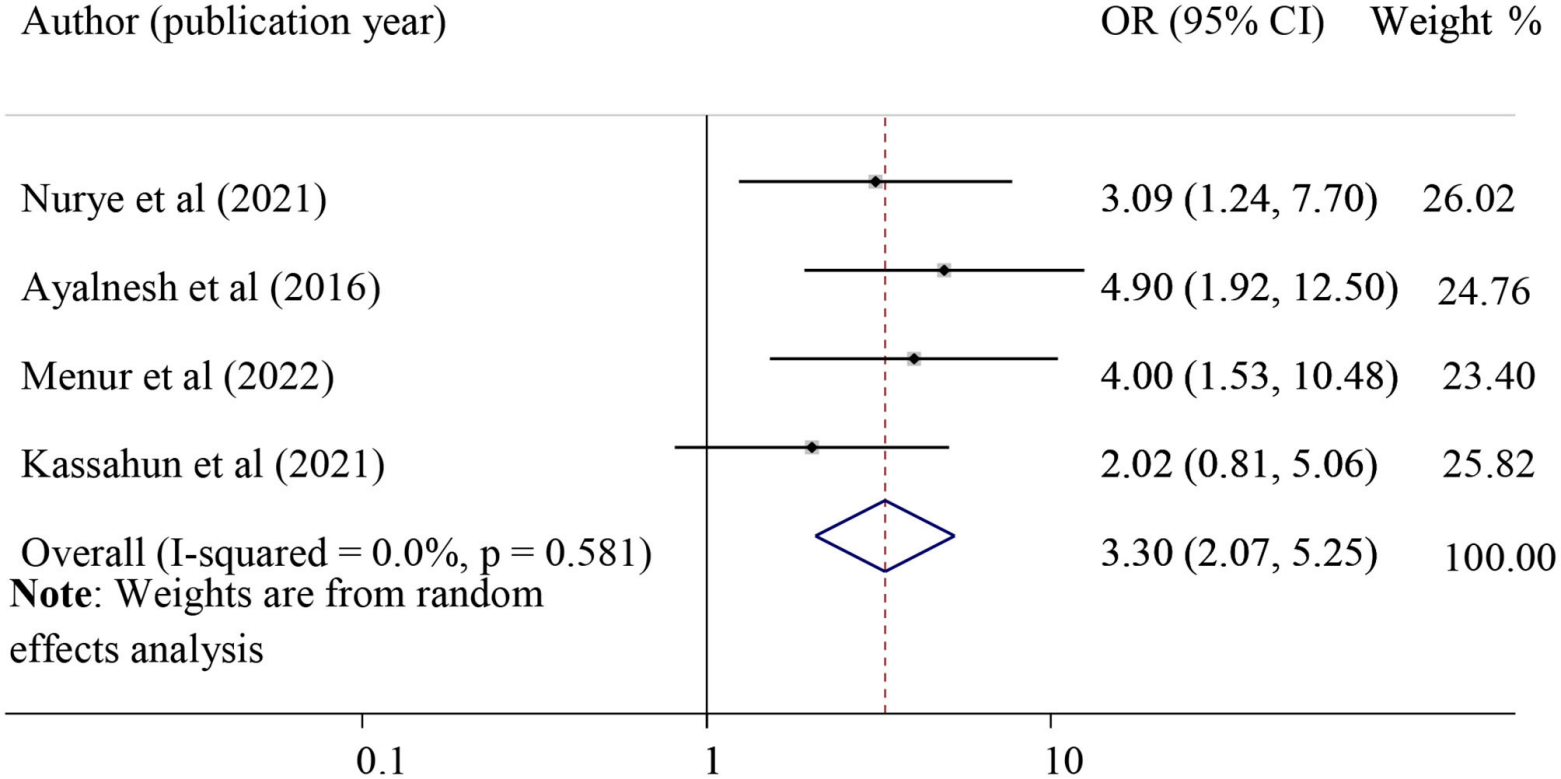
Forest plot for the association of family history with pelvic organ prolapse. OR, odds ratio.

The effect of family history on POP was examined by using four studies (30–32, 42). Those women who had a family history of POP were 3.30 times more likely to develop POP (pooled odds ratio =3.30, 95% CI = 2.07, 5.25) (Figure 7).

#### The association between instrumental delivery and POP

**Figure 8:**
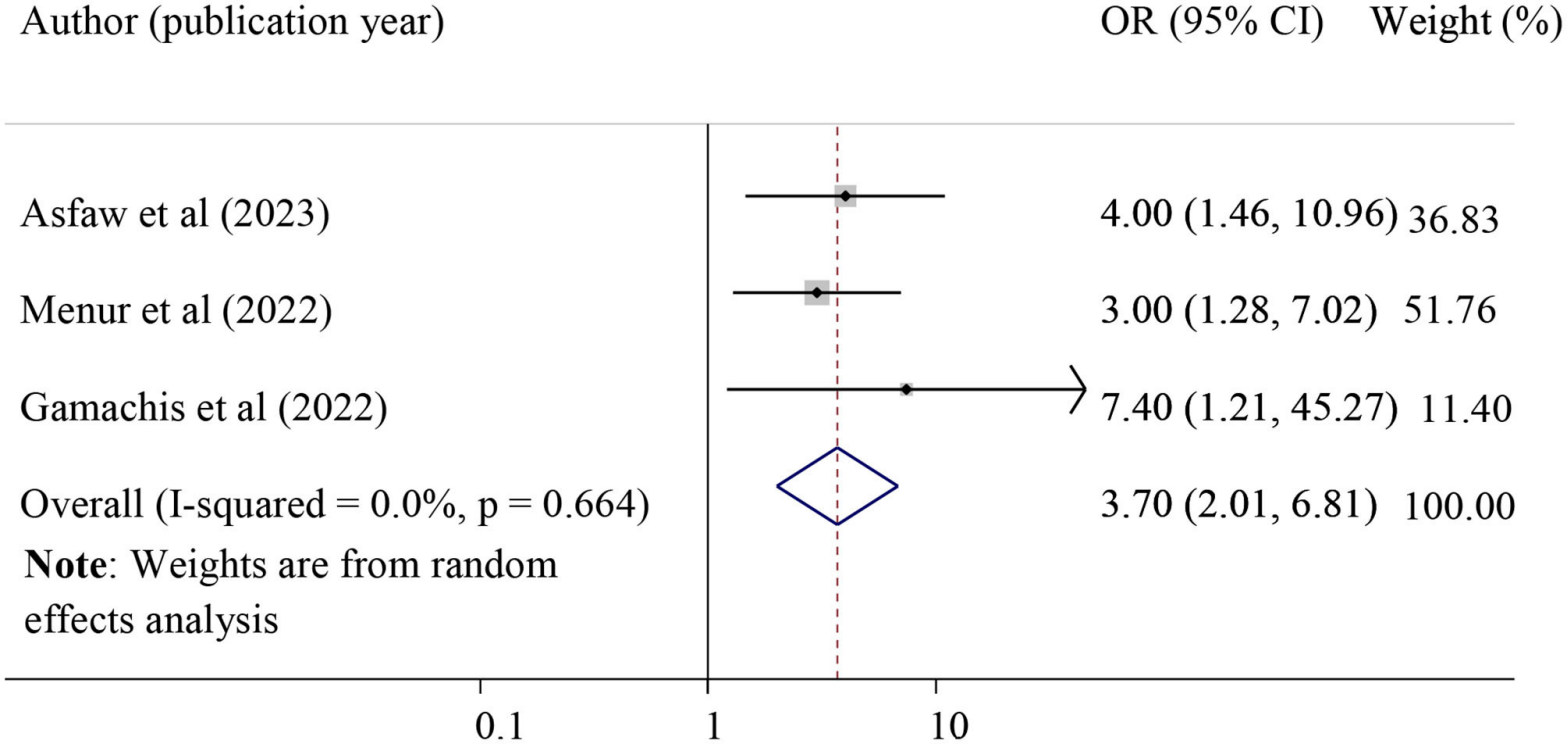
Forest plot for the association of instrumental delivery with pelvic organ prolapse. OR, odds ratio.

The effect of instrumental delivery on POP was examined by using three studies (28, 32, 33). Those mothers who had previous histories of instrumental delivery were 3.70 times more likely to develop POP as compared to those who had a spontaneous vaginal delivery (pooled odds ratio =3.70, 95% CI =2.01, 6.81. In this meta-analysis, instrumental delivery indicates when the women delivered by either using vacuum or forceps delivery at least one time (Figure 8).

#### The association between grand multiparty and POP

**Figure 9:**
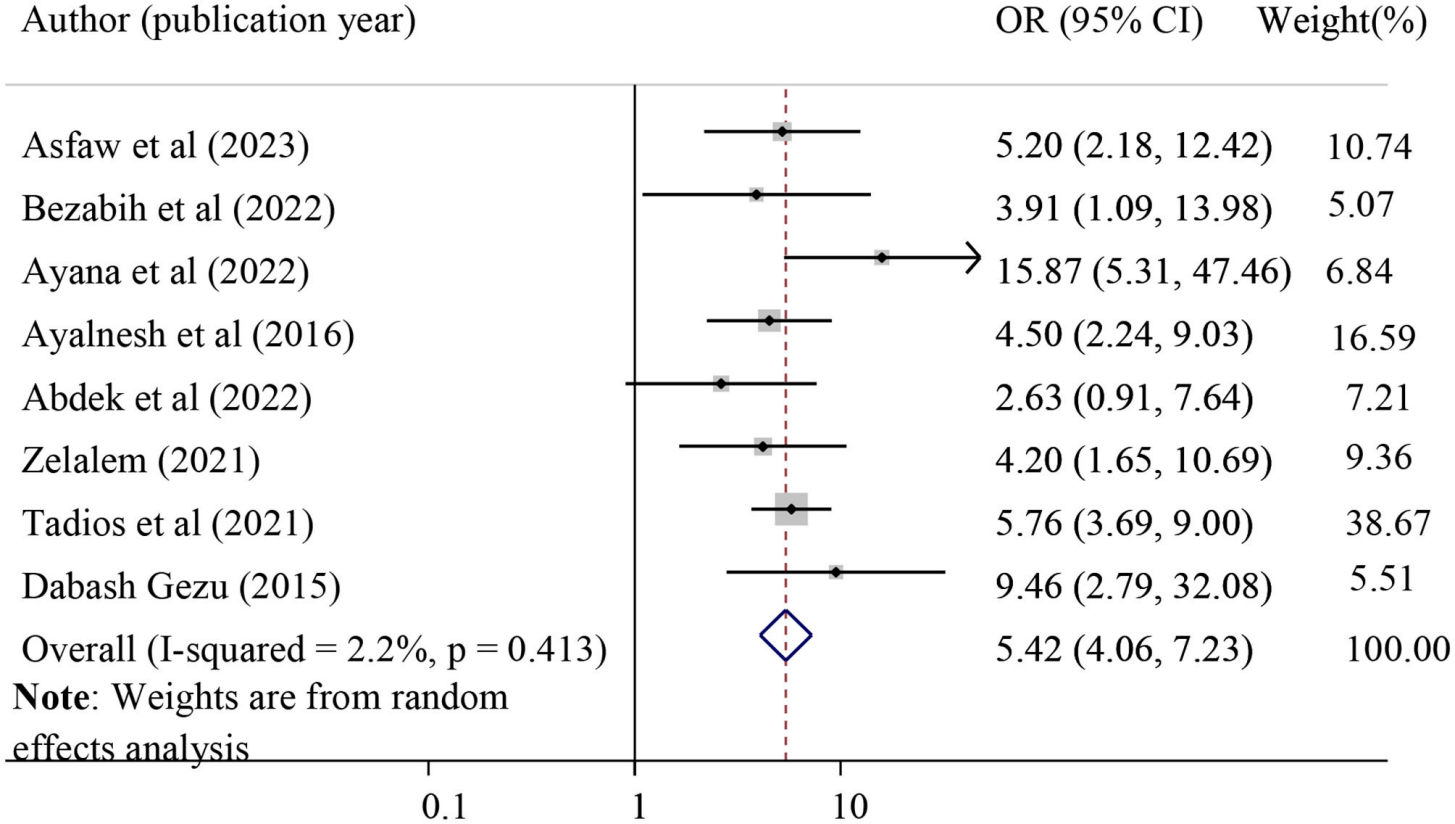
Forest plot for the association of grand multipara with pelvic organ prolapse. OR, odds ratio.

The effect of grand multiparty on POP was evaluated by using eight studies (13, 20, 21, 28, 31, 39, 43, 44), and the result revealed that grand multiparty was significantly associated with POP. Grand multipara mothers were 5.42 times more likely to have POP than those primipara mothers (pooled odds ratio = 5.42, 95%CI = 4.06, 7.23). In this meta-analysis, grand multipara was considered when the women delivered more than or equal to five times (Figure 9).

#### The association between carrying heavy objects and POP

**Figure 10:**
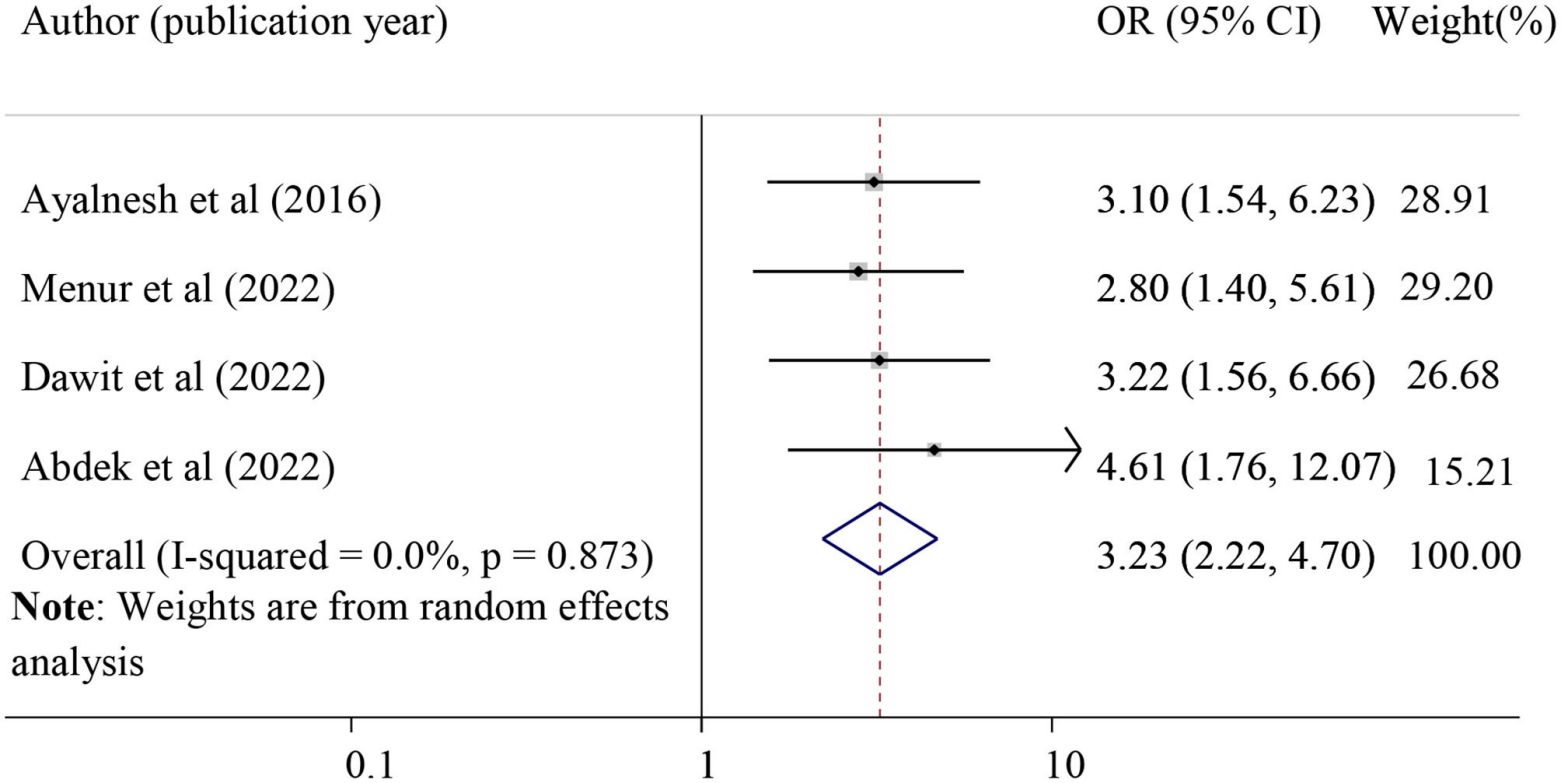
Forest plot for the association of carrying heavy objects with pelvic organ prolapse. OR, odds ratio.

The association between lifting heavy objects and POP was evaluated by using four studies (15, 31, 32, 39). The finding of this study found that lifting heavy objects was significantly associated with POP. Those women who had lifted heavy objects were 3.23 times more likely to have POP (pooled odds ratio = 3.23, 95% CI = 2.22, 4.70). Carrying heavy objects was considered when the woman lifts large objects or performs strenuous physical labor that affects the pelvic organs, such as transporting and selling agricultural goods, gathering wood, and getting water twice per day (Figure 10).

**Figure 11:**
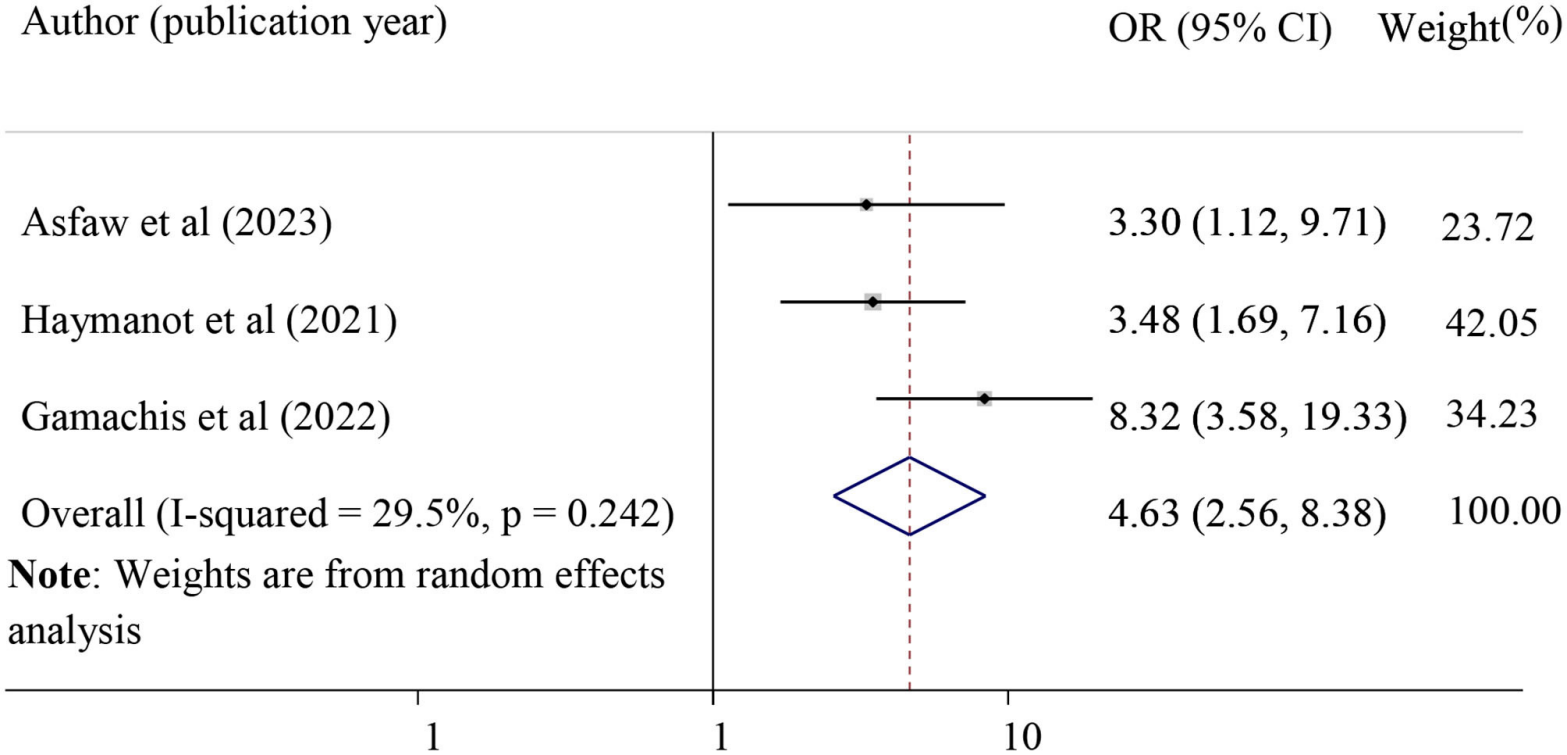
Forest plot shows pooled odds ratio for the association between previous history of prolonged duration of labor and POP. OR, odds ratio.

Finally, we found a strong association between POP and the duration of labor during the last pregnancy, which was evaluated using three studies (28, 33, 37). Those mothers who had prolonged labor were 4.63 times more likely to develop POP as compared to mothers who had the labor of normal duration (pooled odds ratio = 4.63, 95% CI = 2.56, 8.38). In this meta-anlysis, prolonged labor was considered when the total duration of labor is more than 24 h (28, 33, 37) (Figure 11).

## Discussion

The pooled prevalence of POP in Ethiopia was found to be 22.70 with a 95% CI of 16.35 and 29.06. This finding was in line with the finding of a systematic review and meta-analysis carried out in low and middle-income countries, which was 19.7% (45). However, the finding of this meta-analysis study was higher than the study's finding in Nepal (8%) (8), Pakistan (10.3%) (2), Bangladesh (15.6%) (9), South Korea (0.071%) (46), and China (9.6%) (10). A higher burden of POP in this study could be explained by a comparatively higher number of contributing factors for POP, such as a higher prevalence of home deliveries, difficult access to skilled birth delivery, the prolonged second stage of labor, instrumental delivery, and heavy physical workload in Ethiopia. Furthermore, there is a higher fertility rate and early marriages accompanied by many vaginal deliveries in Ethiopia, which are significant predisposing factors for POP.

This finding was lower than a study finding in Tanzania (64.6%) (6), the United Arab Emirates (29.5%) (47), and Brazil (52.3%) (48). Differences in classifications and diagnosis methods for POP across countries might contribute to this variation. In this study, the majority of the primary studies determined the prevalence of POP based on symptom-based diagnosis, which results in a low prevalence of POP. Symptom-based diagnosis might miss asymptomatic POP and may not reflect the exact prevalence of POP. Moreover, the significant variation in the prevalence of POP might be attributed to racial differences, especially compared with the United Arab Emirates and Brazil. Evidence revealed that race is the major risk factor for POP, and the risk of symptomatic prolapse was higher in white and Latina women compared with African women (49).

The result of this study revealed that having a home birth for the last child was significantly associated with POP. This finding was in agreement with a study finding in Nigeria, which stated that having unsupervised home delivery was the most common predisposing factor for POP (50). In addition, there is evidence in Tanzania consistent with the current result (6). This might be attributed to the higher risk of prolonged labor and perineal tears in the case of home delivery. Perineal support during childbirth by a skilled birth attendant is important to prevent perineal lacerations or tears. However, in the case of home delivery, the childbirth process is unsupported or unsupervised by a skilled birth attendant. So, the likelihood of sphincter injury as a result of a perineal tear may be high during home delivery. This condition may result in POP as a long-term complication.

A strong association was observed between high parity and POP, where grand multiparous mothers (greater than or equal to five deliveries) were 5.42 times more likely to have POP than those primiparous mothers. This finding was in agreement with a study finding in Uganda (7), Bangladesh (9), and Tanzania (6). This could be a result of the sphincter muscles and ligaments being damaged by repeated pregnancy and birth, which occasionally never fully recover their strength and suppleness. Moreover, there might be excessive stretching and tearing in the case of grand multipara, which finally results in POP.

This study also revealed that having a perineal tear in the last childbirth was 4.83 times more likely to lead to the development of POP. This finding was supported by a study finding in Nepal, which revealed that having vaginal or sphincter damage in the last childbirth was significantly associated with POP (51). This finding was also in agreement with a study finding in Uganda (7). Injury to the perineal muscles might result in the loss of their supporting role and the widening of the genital hiatus, which eventually leads to prolapse of the pelvic organs.

A significant association was observed between carrying out heavy work and POP. Those women who had lifted heavy objects were 3.23 times more likely to have POP. Evidence in Tanzania supports the current finding that women who carried out heavy work for 5 h or longer each day had a nearly 5-fold higher risk of developing severe POP (6). The possible justification could be due to the fact that lifting large objects or performing strenuous physical labor affects pelvic supporting structures (6).

In this study, the history of instrumental delivery was associated with POP. Evidence from a previous retrospective observational study supports the current finding (52). The greater risk of pelvic floor muscle injuries following instrumental delivery may help to explain why POP is more common in mothers with a history of instrumental delivery. It is a known fact that instrumental delivery is associated with muscle trauma. However, this finding was not supported by a recent systematic review and meta-analysis, which revealed that there was no significant difference in POP between assisted vaginal delivery (including vacuum and forceps) and spontaneous vaginal births (53).

Furthermore, this study also found a strong association between POP and a female family history of POP, where women who had a family history of POP were 3.30 times more likely to develop POP as compared to their counterparts. This finding was in agreement with a systematic review and meta-analysis study, which found that women with at least one female family member with POP have a significantly higher risk of developing this disease (54). Evidence revealed that genetic factors mainly DNA polymorphisms, such as laminin γ1, estrogen receptor α and β, progesterone receptor, collagen, type III, alpha, and matrix metalloproteinase-9, were associated with POP (54).

Finally, we found a strong association between POP and the duration of labor in the last pregnancy. Those mothers who had a prolonged duration of labor were 4.63 times more likely to develop POP as compared to mothers who had a normal duration of labor. This finding was in agreement with a study finding in Uganda that women with prolonged labor in their first delivery were 3.9 times more likely to have POP (7). This might be explained by the possibility of pelvic floor damage in the case of prolonged labor, which subsequently leads to POP as a long-term complication. When the fetal head applies pressure to the pelvic floor for prolonged periods during its engagement in the birth cannula, the pelvic floor muscle, tissue, nerve, and other supporting structures will be damaged. In addition, while the fetus is moving during birth, the levator ani muscle is stretched, causing the hiatus to open. This condition may result in the downward displacement of the pelvic organs from their normal position (55).

## Limitations

This study should be interpreted in light of the following limitations. The presence of heterogeneity and publication bias across studies might affect the generalization of this study. The possible source of heterogeneity was not detected despite performing sub-group analysis and sensitivity analysis.

## Conclusion

In this updated systematic review and meta-analysis, we included a comprehensive number of studies with better geographic coverage than in the prior review. The pooled prevalence of POP was high in Ethiopia. A history of home delivery, prolonged labor, perineal tears, instrumental delivery, grand multipara, a family history of POP, and carrying heavy objects was associated with POP. The result of this study may improve clinicians' and patients' understanding of the individual risk factors for POP. The Ministry of Health and clinicians should emphasize counseling or health education on risk factors and develop further prevention strategies for those modifiable risk factors. Future research should assess the adverse effects or impacts of POP on the quality of life.

## Data availability statement

The original contributions presented in the study are included in the article/Supplementary material, further inquiries can be directed to the corresponding author.

## Author contributions

DA has participated in conceptualization, formal analysis, methodology, data extraction, and writing the original draft. MM, YB, AD, and NG have participated in data curation, formal analysis, methodology, software, and manuscript writing—review and editing. All authors read and approved the final manuscript.
