# Supplementary material for: The prevalence of pelvic organ prolapse and associated factors in Ethiopia: a systematic review and meta-analysis
Source: Front Med (Lausanne). 2023 Jul 5;10:1193069. doi: 10.3389/fmed.2023.1193069 (PMC10354282; doi:10.3389/fmed.2023.1193069)
Supplement: Supplementary file 2 [file Table_2.DOCX]

**S2 Table:** Searching strategies for some databases to assess the pooled uptake of Non-pneumatic Anti-shock Garment (NASG) for the treatment of postpartum hemorrhage in Ethiopia

| **Databases** | **Searching terms** | **Number of studies** | **Searching period** |
| --- | --- | --- | --- |
| PubMed/ MEDLINE | ("epidemiology"[Subheading] OR "epidemiology"[All Fields] OR "magnitude"[All Fields] OR "Proportion"[All Fields] OR "prevalence"[All Fields] OR "prevalence"[MeSH Terms] ) AND ("pelvic organ prolapse"[MeSH Terms] OR ("pelvic"[All Fields] AND "organ"[All Fields] AND "prolapse"[All Fields]) OR "pelvic organ prolapse"[All Fields]) AND ("associated" [All Fields] AND "factors" [All Fields] OR " determinants"[All Fields] OR "predictors" [All Fields]) AND ("ethiopia"[MeSH Terms] OR "ethiopia"[All Fields]) | 156 | **From 2000 /01/01 to 2023/02/14** |
| Google scholar | ( "prevalence" or "burden" or "proportion" ) and "pelvic organ prolapse" and ("associated factors" or "determinants" or "predictors") and "Ethiopia" | 22 |  |
| ScienceDirect | Pelvic organ prolapse and associated factors and Ethiopia | 69 |  |
| DOJA | Pelvic organ prolapse and associated factors and Ethiopia | 10 |  |
| African Journals Online | (prevalence or burden or proportion) and Pelvic organ prolapse and (associated factors or determinants) and Ethiopia | 7 |  |
| Others sources |  | 5 |  |
| Total searched articles |  | 269 |  |
| Finally, fulfill the eligibility criteria for our review |  | 21 |  |
