# Supplementary material for: The prevalence of pelvic organ prolapse and associated factors in Ethiopia: a systematic review and meta-analysis
Source: Front Med (Lausanne). 2023 Jul 5;10:1193069. doi: 10.3389/fmed.2023.1193069 (PMC10354282; doi:10.3389/fmed.2023.1193069)
Supplement: Supplementary file 4 [file Table_4.DOCX]

**S4 Table: A-leave-out -one sensitivity analysis to estimate the pooled prevalence of pelvic organ prolapse in Ethiopia**

| **Study omitted** | **Estimate** | **[95% Conf. Interval]** |
| --- | --- | --- |
| Ayana et al (2022) | 23.87 | 17.11, 30.63 |
| Andualem Henok (2017) | 23.51 | 16.71, 30.30 |
| Dawit et al (2022) | 23.74 | 16.90, 30.59 |
| Abdek et al (2022) | 23.78 | 16.97, 30.59 |
| Merega et al (2018) | 23.92 | 15.90, 31.94 |
| Tadesse et al (2020) | 20.54 | 15.05, 26.04 |
| Haymanot et al (2021) | 21.45 | 15.07, 27.84 |
| Eskedar et al (2021) | 22.47 | 15.83, 29.10 |
| Kassahun et al (2021) | 23.73 | 16.99, 30.47 |
| Abebe et al (2022) | 24.15 | 17.29, 31.00 |
| Zelalem (2021) | 23.65 | 15.37, 31.94 |
| Menur et al (2012) | 21.34 | 14.84, 27.83 |
| Dabash Gezu (2015) | 18.96 | 13.7124.22 |
| **Combined** | **22.70** | **16.34, 29.06** |
